# Biostimulant action of a plant-derived protein hydrolysate produced through enzymatic hydrolysis

**DOI:** 10.3389/fpls.2014.00448

**Published:** 2014-09-09

**Authors:** Giuseppe Colla, Youssef Rouphael, Renaud Canaguier, Eva Svecova, Mariateresa Cardarelli

**Affiliations:** ^1^Department of Agriculture, Forestry, Nature and Energy, University of TusciaViterbo, Italy; ^2^Department of Agricultural Sciences, University of Naples Federico IIPortici, Italy; ^3^Nixe Laboratory, Sophia Antipolis CedexFrance; ^4^Consiglio per la Ricerca e la Sperimentazione in Agricoltura, Centro di Ricerca per lo Studio delle Relazioni tra Pianta e SuoloRoma, Italy

**Keywords:** bioassay, biostimulant, gibberellin-like activity, indole-3-acetic acid, nitrogen uptake, SPAD index

## Abstract

The aim of this study was to evaluate the biostimulant action (hormone like activity, nitrogen uptake, and growth stimulation) of a plant-derived protein hydrolysate by means of two laboratory bioassays: a corn (*Zea mays* L.) coleoptile elongation rate test (Experiment 1), a rooting test on tomato cuttings (Experiment 2); and two greenhouse experiments: a dwarf pea (*Pisum sativum* L.) growth test (Experiment 3), and a tomato (*Solanum lycopersicum* L.) nitrogen uptake trial (Experiment 4). Protein hydrolysate treatments of corn caused an increase in coleoptile elongation rate when compared to the control, in a dose-dependent fashion, with no significant differences between the concentrations 0.75, 1.5, and 3.0 ml/L, and inodole-3-acetic acid treatment. The auxin-like effect of the protein hydrolysate on corn has been also observed in the rooting experiment of tomato cuttings. The shoot, root dry weight, root length, and root area were significantly higher by 21, 35, 24, and 26%, respectively, in tomato treated plants with the protein hydrolysate at 6 ml/L than untreated plants. In Experiment 3, the application of the protein hydrolysate at all doses (0.375, 0.75, 1.5, and 3.0 ml/L) significantly increased the shoot length of the gibberellin-deficient dwarf pea plants by an average value of 33% in comparison with the control treatment. Increasing the concentration of the protein hydrolysate from 0 to 10 ml/L increased the total dry biomass, SPAD index, and leaf nitrogen content by 20.5, 15, and 21.5%, respectively. Thus the application of plant-derived protein hydrolysate containing amino acids and small peptides elicited a hormone-like activity, enhanced nitrogen uptake and consequently crop performances.

## INTRODUCTION

The growing demand for food, feed, fuel, fiber, and raw materials and the increasing resource depletion and ecosystem degradation impose the use of more sustainable methods in the agriculture production systems. Several organic products called “biostimulants” are now available in the market to make agriculture more sustainable. As defined by European Biostimulant Industry Council (www.biostimulants.eu), “plant biostimulants contain substance(s) and/or micro-organisms whose function when applied to plants or the rhizosphere is to stimulate natural processes to enhance/benefit nutrient uptake, nutrient efficiency, tolerance to abiotic stress, and crop quality.” Kauffman et al. (2007) classified organic biostimulant compounds, into three major groups on the basis of their source and content: humic substances, seaweed extracts, and amino acids containing products. The last group consists of free amino acids and polypeptides obtained through chemical and/or enzymatic hydrolysis of agroindustrial by-products from animal or plant origins or from dedicated biomass crops (Cavani et al., 2006). Many studies (Morales-Payan and Stall, 2003; Parrado et al., 2007; Kowalczyk et al., 2008; Ertani et al., 2009; Gurav and Jadhav, 2013) reported beneficial effects of soil and foliar protein hydrolysates applications on growth, yield and fruit quality of agricultural crops (e.g., corn, banana, papaya, strawberry, red grape). Cerdán et al. (2009) and Ertani et al. (2009) observed that applications of plant-derived protein hydrolysates on corn and tomato plants increased nutrient uptake in particular nitrogen and iron as a result of increased nitrate reductase and glutamine synthetase activities, and Fe(III)-chelate reductase activity, respectively. Other authors have highlighted the positive effect of amino acid-derived biostimulants in plant nutrition as chelating agents (Ashmead et al., 1986). Protein hydrolysates can improve crop tolerance to abiotic stresses as reported by Ertani et al. (2013) who observed that root applications of a plant derived-protein hydrolysate improved salinity tolerance of corn due to a better nitrogen metabolism, and an higher K/Na ratio and proline accumulation in leaves.

Protein hydrolysate could also act as plant growth regulators due to the presence of peptides. Several bioactive peptides produced in a variety of plants have been found to have phytohormone-like activities (Ito et al., 2006; Kondo et al., 2006). Phytosulfokine, systemin, SCR/SP11, and CLE are endogenous plants peptides involved in cell differentiation, protease inhibitor induction, cell division, and the pollen self-incompatibility response (Ryan et al., 2002). Recently, Matsumiya and Kubo (2011) isolated from degraded soybean meal products a peptide having root hair promoting activity in *Brassica rapa* and tomato cuttings. Moreover, Ertani et al. (2009) observed that two protein hydrolysates elicited gibberellin-like activity and a weak auxin-like activity.

Besides the plant biostimulant effects of protein hydrolysates, there are also several studies (Ruiz et al., 2000; Cerdán et al., 2009; Lisiecka et al., 2011) reporting that foliar applications of commercial protein hydrolysate products from animal origin can cause phytotoxicity and plant growth depression. On the contrary, no phytotoxicity and growth depression was observed in tomato plants after foliar applications of plant-derived amino acid (Cerdán et al., 2009). Foliar applications of a commercial animal derived-protein hydrolysate caused necrotic spots on basil leaves while no phytotoxic symptoms and growth depression were observed in basil plants after foliar applications of the commercial plant-derived protein hydrolysate “Trainer” up to 10 times the recommended rate (unpublished data). Growth depression caused by animal derived-protein hydrolysates seems to be related to their higher content in free amino acids (especially small size amino acids like glycine, and proline), and salts (e.g., NaCl) than in plant-derived protein hydrolysates.

Recently, there is a growing concern on the use of animal-derived protein hydrolysates in terms of food safety as demonstrated by the ban of animal-derived protein hydrolysate application on the edible parts of crops in organic farming (European Regulation no. 354/2014). Additional limitations may be imposed on animal derived-protein hydrolysate application in the production of food for vegetarians or people with religious dietary restrictions on the consumption of meat due to the need to exclude any contamination of food with animal derived products.

The development of new plant derived-protein hydrolysates with high plant biostimulant activity has become the focus of much research interest. An enzymatic hydrolysis system (LISIVEG^®^;) was recently developed by Italpollina S.p.A. (Rivoli Veronese, Italy) to produce protein hydrolysate (“Trainer”) containing a high concentration of amino acids and soluble peptides. The aim of this study was to investigate the biostimulant action (hormone like activity, nitrogen uptake and growth enhancement) of the plant-derived protein hydrolysate “Trainer” by means of two laboratory bioassays (a corn coleoptile elongation rate test, a rooting test on tomato cuttings) and two greenhouse experiments (a dwarf pea growth test, and a tomato nitrogen uptake trial).

## MATERIALS AND METHODS

### EXPERIMENT 1: CORN COLEOPTILE ELONGATION RATE TEST

A corn hybrid “P1921” (*Zea mays* L.) purchased from Pioneer Hi-Bred Italia S.r.l., Gadesco Pieve Delmona (CR), Italy, was used in the first laboratory test. Corn seed were surface-sterilized with sodium solution hypochlorite (2%) for 20 min to avoid excessive contamination, then washed with distilled water and sown in a plastic box and placed in a growth chamber (24°C) at Tuscia University, Italy. Corn seedlings, were grown in the dark for one week until coleoptiles were 2–3 cm long. The apical 3–4 mm of the coleoptiles were removed and a segment of standard length of 2 cm was cut from the remaining part. These segments were placed into 10 cm petri dishes containing 20 ml of six test solutions: four increasing concentrations (0.375, 0.75, 1.5, and 3 ml L^-1^) of plant-derived protein hydrolysate “Trainer,” 1.75 mg L^-1^ of inodole-3-acetic acid (IAA), and deionized water. The protein hydrolysate “Trainer,” contained 35.5% of organic matter, 5% of total nitrogen, and 27% of amino acids and soluble peptides. The specific weight of the product was 1.1 kg/L. Total amino acid composition, determined after acid hydrolysis and High-Performance Liquid Chromatographic (HPLC) analysis, was: 4% alanine; 6.5% arginine; 11.3% aspartic acid; 1.4% cysteine; 18.5% glutamic acid; 4.6% glycine; 2.6% histidine; 4.3% isoleucine; 8.1% leucine; 6.7% lysine; 1.4% methionine; 5.5% phenylalanine; 5.3% proline; 6.0% serine; 3.9% thereonine; 1.4% tryptophan; 4.4% tyrosine; 5.6% valine. Four replicates were used for each treatment and the experimental unit consist of 10 petri dishes. The increase in length of corn coleoptile was taken after 48 h in the dark as a measure of auxin-like activity.

### EXPERIMENT 2: ROOTING TEST OF TOMATO CUTTINGS

This bioassay was carried out to evaluate the auxin-like activity by estimating the ability of plant-derived protein hydrolysate “Trainer” to promote initiation of adventitious roots in tomato cuttings. Tomato (*S. lycopersicum* L. cv. Marmande, SAIS Sementi, Cesena, Italy) seeds were surface sterilized using commercial bleach with sodium hypochlorite as the active ingredient at 2% for 20 min. After being raised with sufficient water, the tomato seeds were sown in moist vermiculite:peat-based substrate (1:1 volume ratio) in a germination tray. The growth chamber was programmed to maintain a 12 h photoperiod with corresponding 23°C light/18°C night and 65% relative humidity. The light intensity at the canopy level was 450 μmol/m^2^ s, provided by fluorescent lights. After 35 days, the tomato seedlings at three true leaves stage were cut at the base of the stem. The cuttings were immersed for 5 min in a solution containing 6 ml/L of plant-derived protein hydrolysate “Trainer,” whereas distilled water was used as control. The cuttings were planted in transparent plexiglas boxes containing 8 cm of wetted perlite. The plexiglas boxes were closed to ensure a relative humidity close to saturation (100%). Treatments were arranged in a randomized complete block design with three replications. Each experimental unit consist of a plexiglas box containing 30 cuttings.

After 8 days from planting, tomato cuttings were separated into shoots and roots. All plant tissues were dried in a forced-air oven at 80°C for 72 h for biomass determination. Shoot biomass was equal to the sum of aerial vegetative plant parts (leaves + stems). For the root morphology determination, five cuttings per experimental unit were selected. The roots were kindly washing with distilled water, until the root systems were free from any perlite particles. The determination of the root system morphology was done using a WinRHIZO Pro (Regent Instruments Inc., Canada), connected to a STD4800 scanner. Three dimensional images were acquired. The following root characteristics were determined: total root length (mm), mean root diameter (mm) and total root surface area (cm^2^).

### EXPERIMENT 3: DWARF PEA STEM ELONGATION RATE

A tall (“Alderman”) and a dwarf cultivar (“Zaffiro”) of pea (*P. sativum* L.), purchased from Hortus Sementi srl, Longiano, Italy, were used in this bioassay. Seeds were soaked in distilled water for 2–3 h and sown at a rate of three seeds per pots (diameter 6 cm) containing a commercial peat moss based substrate (Brill, Gebr. Brill Substrate GmbH & Co., Georgsdorf, Germany) in a 300 m^2^ polyethylene greenhouse located at the Tuscia University Experimental Farm, Central Italy (42°25′ N, 12°08′ E). Daily temperature was maintained between 18 and 26°C. Night temperature was always greater than 14°C, and relative humidity ranged from 50 to 85%. Treatments were arranged in a randomized complete block design with five replicates. Each experimental unit consisted of 15 plants. Experimental treatments were six different solutions: four increasing concentrations (0.375, 0.75, 1.5, and 3.0 ml/L) of plant-derived protein hydrolysate “Trainer” 100 mg L^-1^ of gibberellic acid (GA_3_), and deionized water (control). Eight days after germination, a drop of the test solutions containing 0.05% of Tween 20 surfactant solution were applied to the shoot of both tall and dwarf pea plants. The application of the solution was repeated 3 days later. Two weeks after sowing stem length was measured on tall and dwarf pea cultivars.

### EXPERIMENT 4: NITROGEN UPTAKE IN TOMATO PLANTS

The fourth experiment was also conducted under greenhouse conditions, in spring 2013. Daily temperature was maintained between 20 and 30°C. Night temperature was always greater than 16°C, and relative humidity ranged from 55 to 85%. *S. lycopersicum* L. cv. Console (SAIS, Seed company, Cesena, Italy) were transplanted on April 3 into pots (diameter 14 cm, height 12 cm) containing 1.5 L of quartziferous sand. The pots were placed on 16 cm wide and 5 m long troughs, with 30 cm between pots and 30 cm between troughs, giving a plant density of 11 plants m^-2^. Treatments were arranged in a randomized complete block design with four replicates. Each experimental unit consisted of fifteen plants. Treatments consisted of three concentrations of plant-derived protein hydrolysate “Trainer” (0, 5, or 10 ml L^-1^). Treatment solutions were applied three times during the growing cycle (April 12, 20, and 26) using a volume of 50 ml/pot. Tomato plants were fertigated daily with a half strength Hoagland solution. All chemicals used were of analytical grade, and composition of the nutrient solution was: 8.0 mM N–${NO}_{3}^{-}$, 1.0 mM S, 0.7 mM P, 2.5 mM K, 3.0 mM Ca, 0.7 mM Mg, 1 mM ${NH}_{4}^{+}$, 20 μM Fe, 9 μM Mn, 0.3 μM Cu, 1.6 μM Zn, 20 μM B, and 0.3 μM Mo, with an electrical conductivity (EC) of 1.2 dS m^-1^. Irrigation scheduling was performed using electronic low-tension tensiometers (LT-Irrometer, Riverside, CA, USA) that controlled irrigation based on substrate matric potential (Rouphael et al., 2004; Rouphael and Colla, 2005).

At the end of the experiment (28 days after transplanting, April 30) a chlorophyll meter (SPAD-502, Minolta corporation, Ltd., Osaka, Japan) was used to take readings from the fully expanded functional leaves. Measurements were made at a central point on the leaflet between midrib and leaf margin. Twenty leaves were measured randomly per plot and averaged to a single SPAD value for each treatment. At the same date of the SPAD measurements, the transplants of tomato were separated into stems, leaves, and roots. All plant tissues were dried in a forced-air oven at 80°C for 72 h for biomass determination. Shoot biomass was equal to the sum of aerial vegetative plant parts (leaves + stems). The dried leaf tissues were ground in a Wiley mill to pass through a 20-mesh screen, then 0.5 g samples were analyzed for the nitrogen content. Nitrogen was determined by the Kjeldahl method (Bremner, 1965) after mineralization with H_2_SO_4_.

### STATISTICAL ANALYSIS

In all experiments, ANOVA tests were conducted using the software package SPSS 10 for Windows (SAS Inc., Cary, NC, USA). Duncan’s multiple range test was performed at *P* = 0.05 on each of the significant variables measured.

## RESULTS AND DISCUSSION

### AUXIN-LIKE ACTIVITY

**FIGURE 1:**
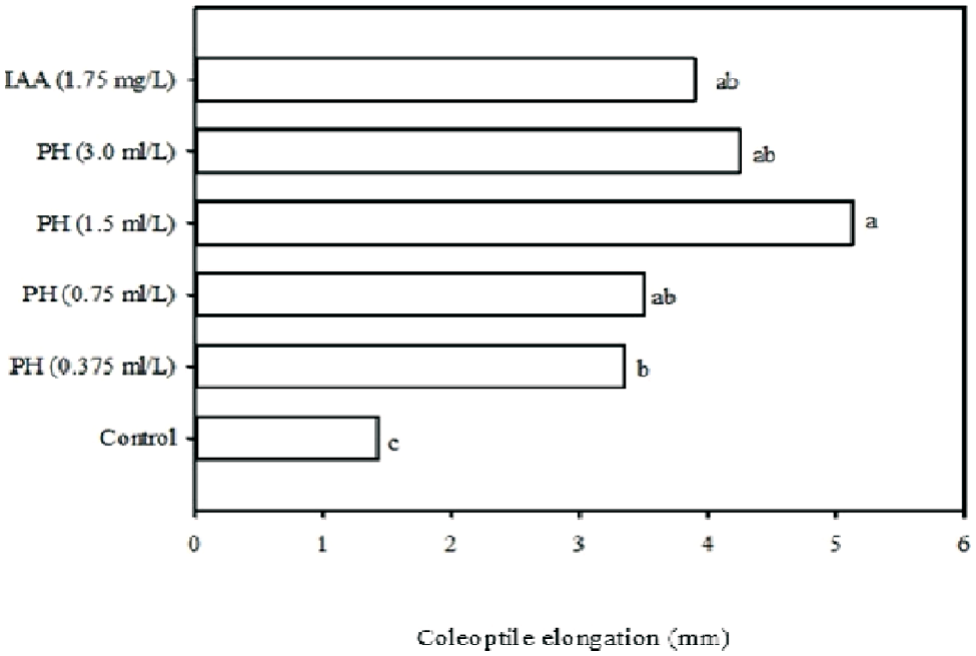
**Corn coleoptile elongation rate in six different solutions: four increasing concentrations (0.375, 0.75, 1.5, and 3.0 ml/L) of plant-derived protein hydrolysate “Trainer” (PH), 1.75 mg/L of inodole-3-acetic acid (IAA), and deionized water (control).** Different letters indicate significant differences according to Duncan’s test (*P* = 0.05). Values are the means of three replicate samples.

It is well established that auxins stimulate stem and cell elongation rate, induce root growth and apical dominance, delay fruit ripening, stimulate fruit development and growth of flowering parts (Zerony and Hall, 1980; Cohen and Bandurski, 1982; Parrado et al., 2008). The auxin-like activity was checked by evaluating the effect of the protein hydrolysate on the corn coleoptile elongation rate which is a typical bioassay for auxins (Audus, 1972). Results of Experiment 1, showed that the type of treatment and concentration significantly (*P* ≤ 0.05) affected the corn coleoptile elongation rate after 48 h of incubation under dark conditions. In this study, the application of 1.75 mg/L of IAA led to an increase of 272% in coleoptile elongation rate when compared to the control. The responses, induced in terms of auxin-like activity (**Figure 1**), showed that the treatment of corn with the protein hydrolysate caused an increase in coleoptile elongation rate when compared to the control, in a dose-dependent fashion, comparable with the effects of IAA, since no significant differences were observed between the four concentrations tested (e.g., 0.375, 0.75, 1.5, and 3.0 ml/L of Trainer) and IAA treatment (**Figure 1**). These results demonstrated that a strong IAA-like activity occurred using the plant-derived protein hydrolysate “Trainer”. Similarly, other commercial products derived from the enzymatic hydrolysis of animal proteins containing a mixture of amino acids and peptides, have been reported to elicit a weak IAA-like activity (Ertani et al., 2009). The higher IAA-like activity of plant-derived protein hydrolysate “Trainer” could be explained by its tryptophan content (1.4%) that is the main precursor for IAA biosynthesis pathways in plants. Moreover, the biological action of peptides may also have contributed to the IAA-like activity of the plant-derived protein hydrolysate. Many secretory and non-secretory peptide signals are involved in various aspects of plant growth regulation including, root and callus growth, defense responses and meristem organization (Matsubayashi and Sakagami, 2006; Ertani et al., 2009).

**FIGURE 2:**
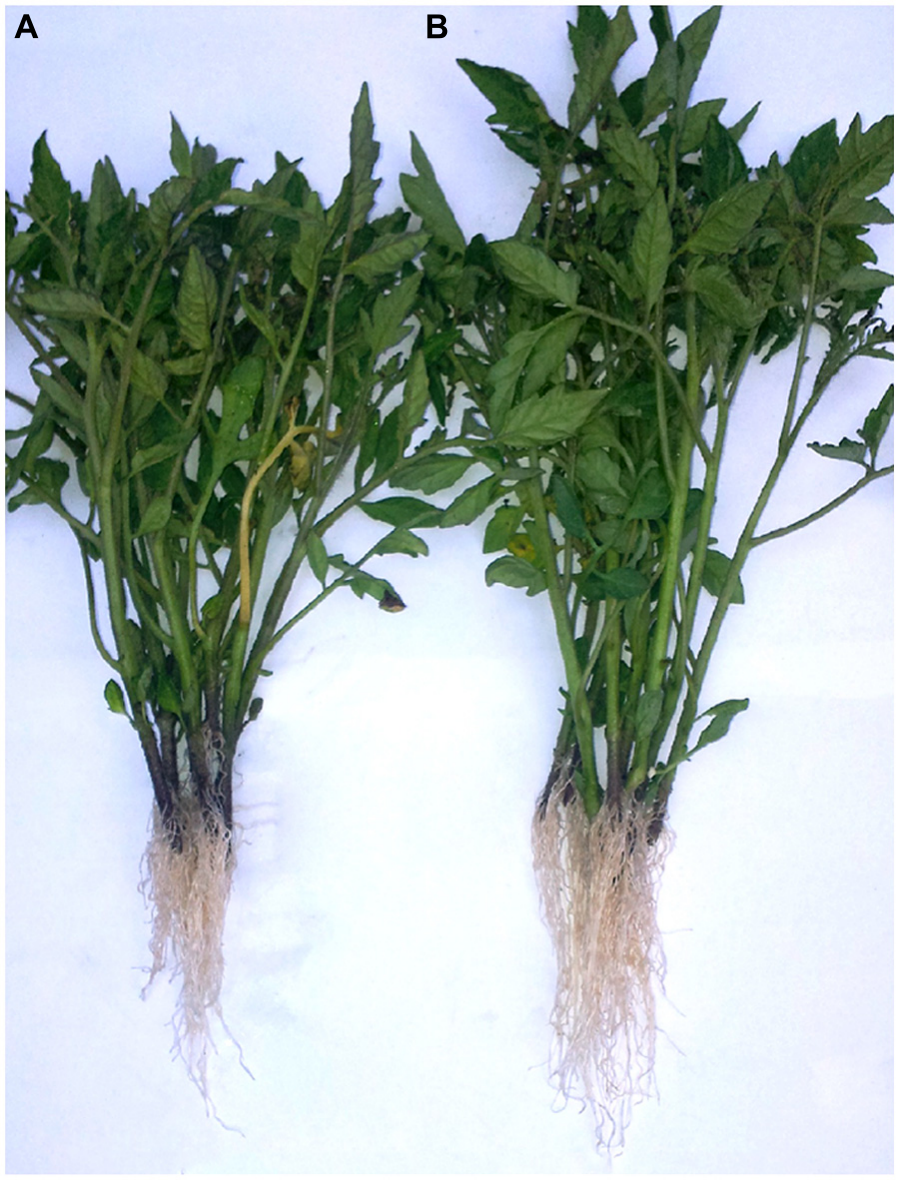
**Tomato cuttings treated with 0 **(A)** and 6 ml/L **(B)** of plant-derived protein hydrolysate “Trainer” at end of the Experiment 2**.

The auxin-like effect of the plant-derived protein hydrolysate “Trainer” has been also observed in the rooting experiment of tomato cuttings (Experiment 2), since treated cuttings with 6 ml/L of the plant-derived protein hydrolysate showed that rooting and shoot growth were stimulated (**Figure 2**). The shoot, root dry weight, root length, and root area were significantly higher by 21, 35, 24, and 26%, respectively, in treated biostimulant than untreated plants (**Table 1**). A stronger and more extensive root apparatus may improve nutrient and water uptake efficiency, leading to an overall increase of plant biomass productivity, and consequently to better yields (Zhang et al., 2003; Ertani et al., 2009). In a recent study, Matsumiya and Kubo (2011) identified a root hair promoting peptide from a degraded soybean meal product. The degraded soybean meal product containing the root hair promoting peptide increased the number of root hairs of *B. oleracea* L., *Lactuca sativa*, *Trifolium incarnatum* L., and *Gypsophila elegans*. The presence of root hairs are important root parts for the absorption and transport of nutrients (Lauter et al., 1996; Gilroy and Jones, 2000). Matsumiya and Kubo (2011) concluded that the enhancement of plant growth by degraded soybean meal products was caused by the increase of root hair numbers and length. HPLC analysis revealed the presence of the root hair promoting peptide in the tested plant-derived protein hydrolysate (1 g/L).

**Table 1:** Effects of plant-derived protein hydrolysate application on dry weight of shoots and roots, total root length, total root surface, and average root diameter of tomato cuttings.

Protein hydrolysate (ml L^-1^)	Shoot dry weight (mg/plant)	Root dry weight (mg/plant)	Root length (cm/plant)	Root surface (cm^2^/plant)	Root mean diameter (mm)
0	361.0	20.1	150.1	17.3	0.36
6	437.0	27.1	186.3	21.8	0.37
Significance	**	**	**	*	ns

ns, *^,^**non-significant or significant at *P* ≤ 0.05, and 0.01, respectively.

### GIBBERELLIN-LIKE ACTIVITY

**FIGURE 3:**
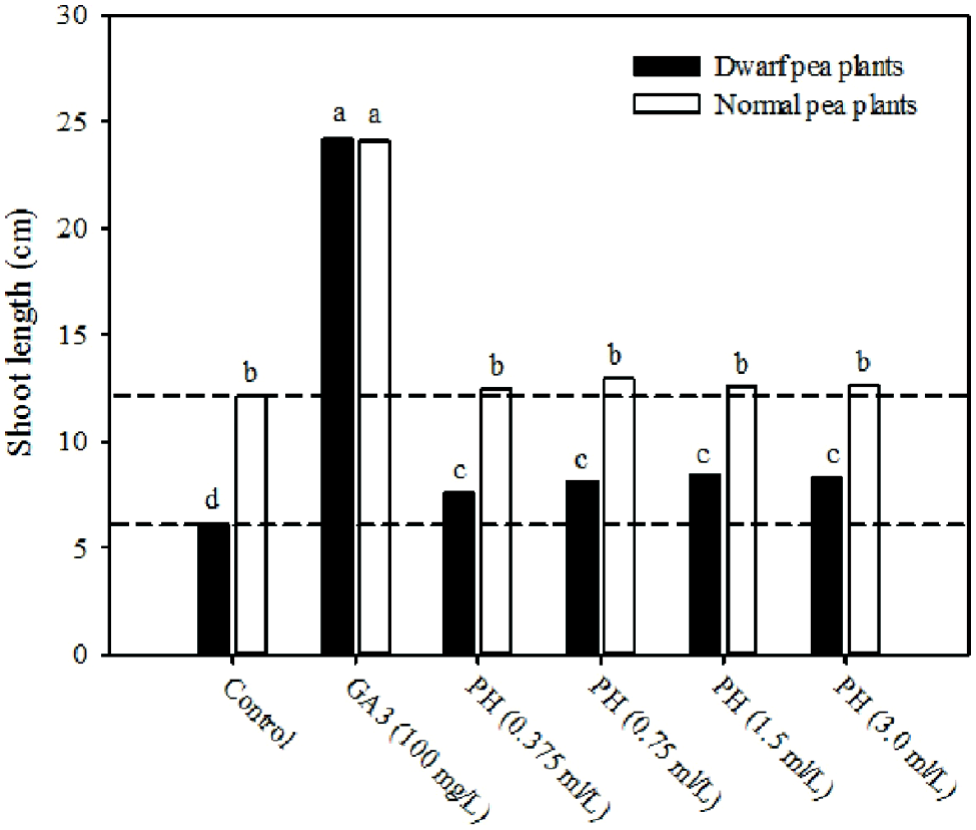
**Shoot length of normal and dwarf pea plants treated with six different solutions: four increasing concentrations (0.375, 0.75, 1.5, and 3.0 ml/L) of plant-derived protein hydrolysate “Trainer” (PH), 100 mg/L of gibberellic acid (GA_**3**_), and deionized water (control).** Dashed lines indicate shoot length of normal and dwarf pea plants in control treatment. Different letters indicate significant differences according to Duncan’s test (*P* = 0.05). Values are the means of four replicate samples.

Gibberellins (GA_3_), like auxins, promote cell elongation rate, and act as chemical messengers to stimulate the synthesis of enzymes such as α-amylase and other hydrolytic enzymes important during seedlings in order to ensure release of stored nutrients, stimulate leaf growth, flowering, and fruit set (Philipson, 1985; Longman et al., 1986; Parrado et al., 2008). The dwarf pea stem elongation rate test (Experiment 3) was used to assay the gibberellin-like activity of the plant-derived protein hydrolysate “Trainer.” Treatment of dwarf plants with gibberellic acid at a concentration of 100 mg/L increased shoot length to similar values of normal pea plants (**Figure 3**). The application of plant-derived protein hydrolysate “Trainer” at all doses significantly increased the shoot length of the gibberellin (GA)-deficient dwarf pea plants by an average value of 33% in comparison with the control treatment (**Figure 3**), providing additional evidence of a gibberellin-like activity. These results are consistent with the findings of Ertani et al. (2009), who reported that the application of protein hydrolysate-derived products significantly increased the shoot length of lettuce, when compared to the application of GA_3_ indicating a strong gibberellin-like activity.

### GROWTH AND NITROGEN METABOLISM IN TOMATO PLANTS

In Experiment 4, all measured tomato growth parameters were significantly influenced by the plant-derived protein hydrolysate applications. Our data indicated that the two concentrations (5 or 10 ml/L) tested enhanced growth of tomato transplants as evidenced by the shoot (+19.5%), root (+27.5%), and total dry biomass (+20.5%, **Table 2**). In addition, increasing the concentration of the plant-derived protein hydrolysate from 0 to 10 ml/L increased chlorophyll content (SPAD index) and leaf nitrogen content by 15 and 21.5%, respectively. The positive effect exerted by plant-derived protein hydrolysate “Trainer” on plant growth parameters occurred through stimulation of nitrogen uptake and assimilation. Increased leaf nitrogen content may account for enhanced photosynthesis and improved translocation of photosynthates to the sinks that contribute to the greater plant biomass of plants treated with the protein hydrolysate. Furthermore, plant nitrogen assimilation involves the synthesis and conversion of amino acid through the reduction of nitrate. Several key enzymes (e.g., nitrate reductase and glutamine synthetase) are responsible of these processes. It has been reported that root applications of protein hydrolysates can increase nitrogen assimilation through an increase of nitrate reductase and glutamine synthetase activities as previously observed in corn (Ertani et al., 2009). Moreover, the increase of root apparatus resulting from the protein hydrolysate applications may also have contributed to increase nitrogen uptake by tomato plants. The positive effects of protein hydrolysate application on leaf nitrogen content have been also observed on several vegetable crops such as lettuce, radish, and red pepper (Liu and Lee, 2012; Tsouvaltzis et al., 2014).

**Table 2:** Effects of plant-derived protein hydrolysate applications on dry weight of shoots, roots and total biomass, SPAD index, and leaf nitrogen content of tomato plants.

Protein hydrolysate (ml L^-1^)	Dry biomass (g/plant)			SPAD	Leaf N content (g/kg)
	Shoot	Root	Total
0	4.85 b	0.71 b	5.56 b	39.0 b	25.4 b
5	5.52 a	0.92 a	6.44 a	44.3 a	30.1 a
10	6.07 a	0.89 a	6.96 a	45.2 a	31.6 a
Significance	**	**	**	*	**

*,** significant at *P* ≤ 0.05, and 0.01, respectively. Different letters within each column indicate significant differences according to Duncan’s multiple-range test (*P* = 0.05).

## CONCLUSION

In summary, the present study demonstrated the biostimulant effects of the plant-derived protein hydrolysate “Trainer” on growth parameters of corn, pea, and tomato. The application of plant-derived protein hydrolysate “Trainer” elicited an auxin and gibberellin-like activity, enhanced nitrogen uptake, and crop performance. The high nitrogen uptake observed in plants treated with “Trainer” could be explained by the extensive root apparatus and the increase of nitrogen assimilation process. Finally, the use of this product could be of practical interest for promoting plant growth and reducing nitrogen fertilizers because it can increase nitrogen use efficiency. However, further researches are required for understanding the action mechanisms of the biostimulation on plants.

## Conflict of Interest Statement

The authors declare that the research was conducted in the absence of any commercial or financial relationships that could be construed as a potential conflict of interest.
